# PRRSV GP2a blocks the RLR signaling pathway by targeting RIG-I

**DOI:** 10.1128/jvi.01135-25

**Published:** 2025-10-10

**Authors:** Yingjie Xiang, Chunxiao Mou, Xing Zhao, Chen Zhuo, Kaichuang Shi, Yun Young Go, Zhenhai Chen

**Affiliations:** 1 College of Veterinary Medicine, Yangzhou University 38043, Yangzhou, China; 2 Jiangsu Co-Innovation Center for Prevention and Control of Important Animal Infectious Diseases and Zoonoses, Yangzhou University 38043, Yangzhou, China; 3 Guangxi Center for Animal Disease Control and Prevention, Nanning, Guangxi, China; 4 College of Veterinary Medicine, Konkuk University 34965, Seoul, Republic of Korea; 5 Joint International Research Laboratory of Agriculture and Agri-Product Safety, the Ministry of Education of China, Yangzhou University 38043, Yangzhou, China; University of North Carolina at Chapel Hill, Chapel Hill, North Carolina, USA

**Keywords:** PRRSV, GP2a, RIG-I, RNF125, TRIM25, ZCCHC3

## Abstract

The strategy of viruses targeting RIG-I to disrupt the interferon (IFN) system represents an effective mechanism for evading innate immune responses. In this study, we observed that porcine reproductive and respiratory syndrome virus (PRRSV) GP2a inhibited IFN production by targeting RIG-I. Further studies revealed that GP2a blocks the RIG-I-like receptor signaling pathway through two mechanisms: (i) GP2a induces K48-linked ubiquitination of RIG-I by promoting the interaction between RIG-I and RING finger protein 125 (RNF125), resulting in RIG-I degradation, and (ii) GP2a hijacks zinc finger CCHC-type containing 3 (ZCCHC3) to disrupt the formation of the tripartite motif-containing 25 (TRIM25)-RIG-I complex, thereby inhibiting RIG-I K63-linked ubiquitination. This inhibition effectively prevents the activation and expression of RIG-I. In conclusion, our findings demonstrate novel mechanisms by which PRRSV GP2a inhibits IFN production, thereby improving our understanding of PRRSV immune evasion strategies.

**IMPORTANCE:**

Porcine reproductive and respiratory syndrome is an important viral disease that affects the swine industry worldwide. PRRSV glycoproteins (GPs) play a crucial role in the viral infection process. However, it remains largely unknown about what roles PRRSV GPs play in antagonizing the innate immune response. In this study, we found that GP2a targets RIG-I to inhibit IFN production through a dual-faceted mechanism. GP2a promotes the RNF125-mediated degradation of RIG-I and competitively interacts with ZCCHC3 to impede TRIM25-induced RIG-I activation. This research contributes to a deeper understanding of the immune escape mechanisms employed by PRRSV.

## INTRODUCTION

Porcine reproductive and respiratory syndrome (PRRS) was first identified in 1987 and has rapidly disseminated worldwide, resulting in significant economic losses to the global swine industry (1, 2). PRRS is caused by the porcine reproductive and respiratory syndrome virus (PRRSV), which is characterized by respiratory and reproductive disorders in sows and is responsible for piglet mortality (1). PRRSV belongs to the order *Nidovirales*, the family *Arteriviridae*, and the genus *Betaarterivirus* (3). It is a single-stranded positive-sense enveloped RNA virus with a genome size of approximately 15 kb (3, 4). The PRRSV genome contains at least 10 open reading frames encoding 16 nonstructural proteins and eight structural proteins, including glycoproteins (GPs), a matrix protein (M), and a nucleocapsid protein (N) (4). GP2a forms heterotrimeric complexes with GP3 and GP4 to interact with CD163, facilitating PRRSV entry into host cells and thus playing a crucial role in PRRSV infection (5). GP5 and M proteins form heterotrimeric complexes that play important roles in viral attachment (6). The N protein encapsulates the viral genome within nucleocapsids, a process that is essential for virion assembly (7).

The interferon (IFN) system is a crucial component of the host’s innate immune response, serving as the first line of defense against viral infections (8, 9). After viral infection, retinoic acid-inducible gene I (RIG-I) undergoes conformational changes to recognize the viral RNA (10). Subsequently, RIG-I interacts with mitochondrial antiviral signaling protein (MAVS) to recruit downstream signaling molecules, such as TANK-binding kinase 1 (TBK1) and inhibitor of NF-κB kinase-ε (IKKε), thereby inducing phosphorylation of IFN regulatory factor 3 (IRF3) and IRF7 (11, 12). Once phosphorylated, IRF3 and IRF7 translocate to the nucleus and associate with CREB-binding protein (CBP) to form transcriptional enhancers that stimulate IFN transcription (13, 14). Subsequently, IFN activates the Janus kinase-signal transducer and activator of the transcription (JAK-STAT) signaling pathway, leading to the production of interferon-stimulated genes (ISGs) (15). Notably, RIG-I is an ISG that establishes a positive feedback loop, further increasing RIG-I production (16). It is worth noting that the ubiquitin (Ub) system plays an important role in regulating RIG-I activation (17). E3 Ub ligases, such as tripartite motif-containing 25 (TRIM25), positively regulate RIG-I by inducing K63-linked ubiquitination (18). This modification can promote RIG-I activation, thereby promoting IFN production (18). Conversely, some E3 Ub ligases function as negative regulators of RIG-I. RING finger protein 125 (RNF125) and tripartite motif-containing 40 (TRIM40) promote RIG-I degradation by inducing its K48-linked ubiquitination, ultimately inhibiting IFN production (19, 20). Furthermore, RIG-I ubiquitination is modulated by various host factors. For instance, Src homology 3 domain-containing kinase-binding protein 1 (SH3KBP1) and zinc finger CCHC-type containing 3 (ZCCHC3) induce K63-linked polyubiquitination of RIG-I by improving its interaction with TRIM25 (21, 22). Moreover, Ras-GTPase-activating protein (GAP)-binding protein 1 (G3BP1) interacts with RNF125 to promote its degradation, thereby inhibiting RNF125-mediated RIG-I degradation (23).

Previous studies have demonstrated that PRRSV develops various strategies to evade host innate immune defenses by disrupting the IFN system (24). For instance, PRRSV nsp1α inhibits IFN production by interacting with TRIM25, thereby preventing RIG-I activation (25). Furthermore, nsp4 blocks the NF-κB signaling pathway by cleaving the NF-κB essential modulator (NEMO). Nsp11 inhibits the formation and nuclear translocation of interferon-stimulated gene factor 3 (ISGF3) by targeting interferon regulatory factor 9 (IRF9) (26). Moreover, nsp11 degrades ISG15 through endoribonuclease activity (27). The N protein interferes with RIG-I activation by targeting TRIM25 and inhibits IRF3 activation by preventing its phosphorylation and nuclear translocation (28). Recent studies have identified that PRRSV GPs also play a role in immune evasion. Specifically, GP3 inhibits IFN production by blocking phosphorylation of TBK1 and IRF3 (29). GP5 inhibits chaperone-mediated autophagy by blocking K63-linked polyubiquitination of lysosome-associated membrane GP 2 (LAMP2A), thereby suppressing IFN production (30). However, it remains uncertain whether GP2a is involved in PRRSV immune evasion.

This study demonstrated that GP2a induces RIG-I degradation. However, the specific mechanism by which GP2a induces this degradation remains unknown. Therefore, we attempted to address the following questions. (i) Which pathway does GP2a induce RIG-I degradation through? (ii) What mechanism does GP2a induce RIG-I degradation by? (iii) Does GP2a affect RIG-I ubiquitination? Addressing these issues can help us gain a deeper understanding of the immune evasion mechanisms of PRRSV.

## RESULTS

### PRRSV-induced RIG-I degradation

**Fig 1:**
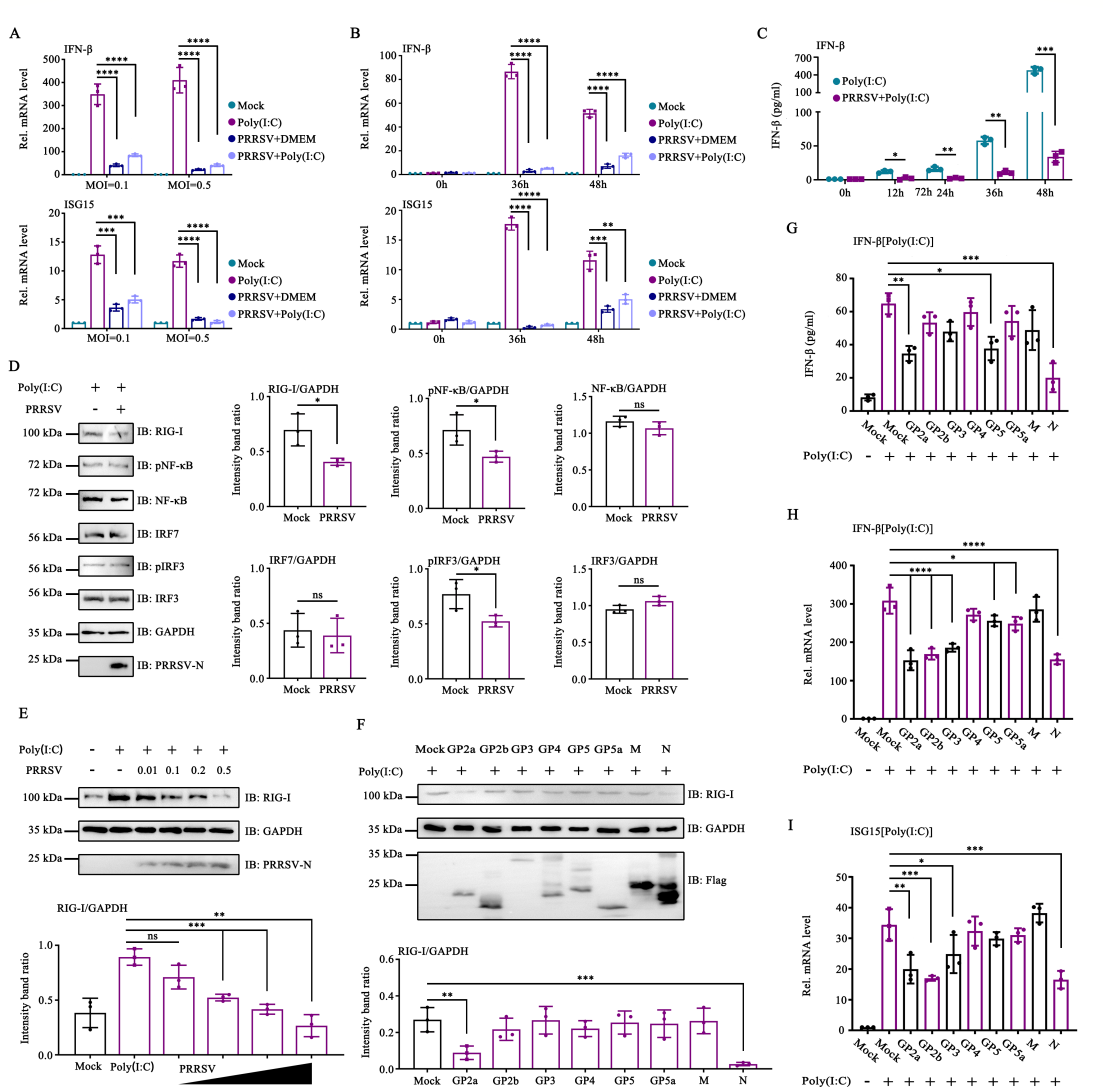
PRRSV-induced RIG-I degradation. (**A**) Marc-145 cells were infected with PRRSV at an MOI of 0.1 or 0.5. IFN-β and ISG15 mRNA levels were determined by RT-qPCR. (**B and C**) Marc-145 cells were infected with PRRSV at an MOI of 0.1. After 24 hpi, cells were treated with Poly(I:C). (**B**) After 12 or 24 h, the cells were collected to detect IFN-β and ISG15 mRNA levels using RT-qPCR. (**C**) At 0, 12, 24, 36, 48, 60, and 72 hpi, cell supernatants were collected to detect IFN-β using ELISA. (**D**) Marc-145 cells were infected with PRRSV at an MOI of 0.1 for 36 h. RIG-I, pNF-κB, NF-κB, pIRF3, IRF3, IRF7, GAPDH, and Flag were detected using Western blot assay. (**E**) Marc-145 cells were infected with PRRSV at an MOI of 0.01, 0.1, 0.2, or 0.5 for 36 h. RIG-I, GAPDH, and Flag were detected by Western blot assay. (**F–I**) HEK-293T cells were transfected with plasmids expressing the PRRSV structural proteins. (**F**) The cells were collected to detect RIG-I, GAPDH, and Flag using Western blot assay. (**G**) Cell supernatants were collected to detect IFN-β using ELISA. (**G–I**). Cells were collected to detect IFN-β, and ISG15 mRNA levels were determined by RT-qPCR. (**A–C and G–I**) The experiment was conducted three times, and the data are shown as mean ± standard deviation (SD) from triplicate wells in the same experiment (one-way ANOVA; *, *P* < 0.05; **, *P* < 0.01; ***, *P* < 0.001; ****, *P* < 0.0001). (**D–F**) Relative band intensities were quantified using ImageJ software. Data are shown as mean ± SD from three independent experiments (Student’s *t*-test for **D**, one-way ANOVA for **E and F**; *, *P* < 0.05; **, *P* < 0.01; ***, *P* < 0.001). ns, not significant.

To determine the effect of PRRSV on IFN production and response, we initially assessed whether PRRSV infection influences the expression of IFN-β and ISG15. Marc-145 cells were infected with PRRSV at varying multiplicities of infection (MOIs), and cell samples were collected to measure the mRNA levels of IFN-β and ISG15. PRRSV did not significantly increase the mRNA levels of IFN-β and ISG15 compared with the Poly(I:C) group (Fig. 1A). PRRSV reduced the expression of IFN-β and ISG15 mRNA in cells treated with Poly(I:C) (Fig. 1A). Further analysis was performed to determine the mRNA levels of IFN-β and ISG15 at different time points post-infection (hpi). PRRSV significantly inhibited the expression of IFN-β and ISG15 mRNA at 36 and 48 hpi (Fig. 1B). To further validate the results, we measured IFN-β levels in the culture supernatant using an enzyme-linked immunosorbent assay (ELISA). Compared to the Poly(I:C) group, the PRRSV infection group demonstrated significantly lower secretion levels of IFN-β at 12, 24, 36, and 48 hpi (Fig. 1C). These findings indicate that PRRSV inhibits IFN production. Subsequently, we investigated the specific targets within the signaling cascade at which PRRSV blocks IFN production. The results revealed that PRRSV significantly inhibited RIG-I expression (Fig. 1D). To validate this finding, Marc-145 cells were infected with PRRSV at various MOIs. The degradation of RIG-I induced by PRRSV became more pronounced with an increase in the infection dose (Fig. 1E). These findings suggested that PRRSV inhibits RIG-I expression to block IFN production.

Previous studies have reported that PRRSV nsp1α, nsp2, nsp5, nsp11, and N inhibit IFN production by targeting RIG-I. However, it remains unclear whether PRRSV GPs are involved in this antagonistic function. HEK-293T cells were transfected with plasmids encoding different GPs. Cells transfected with plasmids encoding N proteins served as controls. Western blot analysis revealed that GP2a significantly inhibited RIG-I expression (Fig. 1F). The effect of PRRSV structural proteins on IFN-β expression was detected using ELISA. The results showed that GP2a treatment significantly inhibited IFN-β expression (Fig. 1G). To verify this result, we evaluated the effects of PRRSV GPs on IFN-β and ISG15 mRNA expression levels. GP2a inhibited IFN-β and ISG15 mRNA expression stimulated by Poly(I:C) (Fig. 1H and I). These findings suggest that PRRSV GP2a plays a crucial role in inhibiting IFN production.

### PRRSV GP2a induced RIG-I degradation through the Ub proteasome pathway

**Fig 2:**
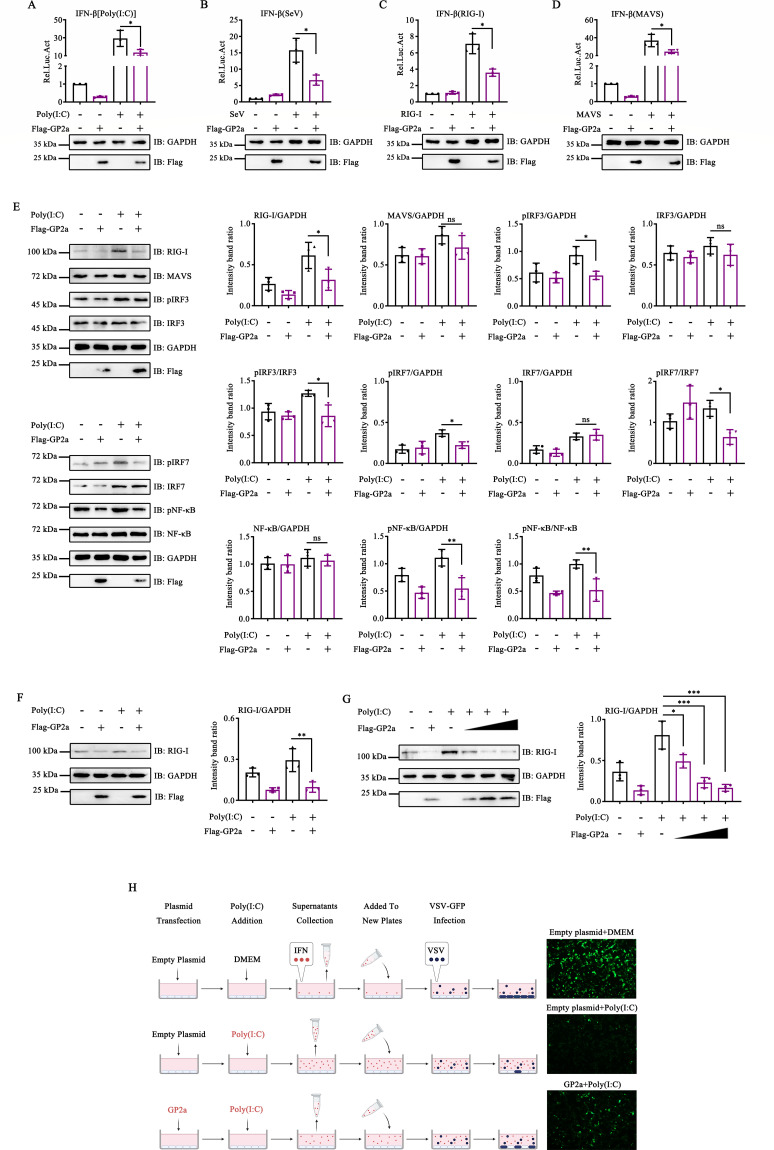
PRRSV GP2a-induced RIG-I degradation through the Ub proteasome pathway. (**A and B**) HEK-293T cells were co-transfected with pCAGGS-3×Flag-GP2a, pIFN-β-luc, and pRL-TK. (**A**) Cells were stimulated with Poly(I:C) for 12 h. (**B**) Cells were stimulated with SeV for 12 h. (**C**) HEK-293T cells were co-transfected with pCAGGS-3×Flag-GP2a, pIFN-β-luc, pRL-TK, and pCDNA3.1-2HA-RIG-I. (**D**) HEK-293T cells were co-transfected with pCAGGS-3×Flag-GP2a, pIFN-β-luc, pRL-TK, and pCDNA3.1-2HA-MAVS. (**A–D**) The cells were collected to detect IFN-β promoter activity using a dual-luciferase reporter assay. The experiment was conducted three times, and the data are shown as mean ± SD from triplicate wells in the same experiment (one-way ANOVA; *, *P* < 0.05). (**E**) HEK-293T cells were transfected with pCAGGS-3×Flag-GP2a. The cells were collected to detect the expression of RIG-I, IRF3, phosphorylated IRF3, NF-κB, phosphorylated NF-κB, IRF7, phosphorylated IRF7, GAPDH, and N proteins using Western blot assay. (**F**) Marc-145 cells were transfected with pCAGGS-3×Flag-GP2a. (**G**) HEK-293T cells were transfected with pCAGGS-3×Flag-GP2a at 250, 500, or 1,000 ng. (**F and G**) The cells were collected to detect RIG-I, GAPDH, and Flag using Western blot assay. (**H**). HEK-293T cells were transfected with pCAGGS-3×Flag-GP2a and treated with Poly(I:C). Cell supernatants were collected and added to new plates. Fluorescence was observed after infecting the cells with VSV-GFP. (**E–G**) Relative band intensities were quantified using ImageJ software. Data are shown as mean ± SD from three independent experiments (one-way ANOVA; *, *P* < 0.05; **, *P* < 0.01; ***, *P* < 0.001). ns, not significant.

To elucidate the mechanism by which PRRSV GP2a inhibits IFN production, we investigated the effect of GP2a on the activation of the IFN-β promoter. GP2a inhibited the activation of the IFN-β promoter induced by Poly(I:C) or Sendai virus (SeV) (Fig. 2A and B). Notably, GP2a inhibited the activation of the IFN-β promoter induced by RIG-I, suggesting that GP2a may impede the IFN production through targeting RIG-I (Fig. 2C and D). Subsequent analyses revealed that GP2a induced RIG-I degradation and inhibited phosphorylation of IRF3, IRF7, and NF-κB (Fig. 2E). Similarly, GP2a also induced the degradation of RIG-I in Marc-145 cells (Fig. 2F). To verify these findings, HEK-293T cells were transfected with varying doses of a plasmid encoding GP2a. The results demonstrated that GP2a significantly induced RIG-I degradation in a dose-dependent manner (Fig. 2G). Furthermore, we detected the IFN-β production in the supernatants by analyzing the infection efficiency of vesicular stomatitis virus green fluorescent protein (VSV-GFP). In the empty plasmid + Poly(I:C) group, Poly(I:C) effectively stimulated the cells to produce substantial amounts of IFN-β, resulting in a marked inhibition of VSV-GFP infection. In the Poly(I:C) + GP2a group, GP2a induced the degradation of RIG-I, thereby impairing the ability of Poly(I:C) to induce IFN-β production. The results showed that the infection efficiency of VSV-GFP was enhanced (Fig. 2H).

### PRRSV GP2a promoted K48-linked ubiquitination of RIG-I and inhibited K63-linked ubiquitination of RIG-I

**Fig 3:**
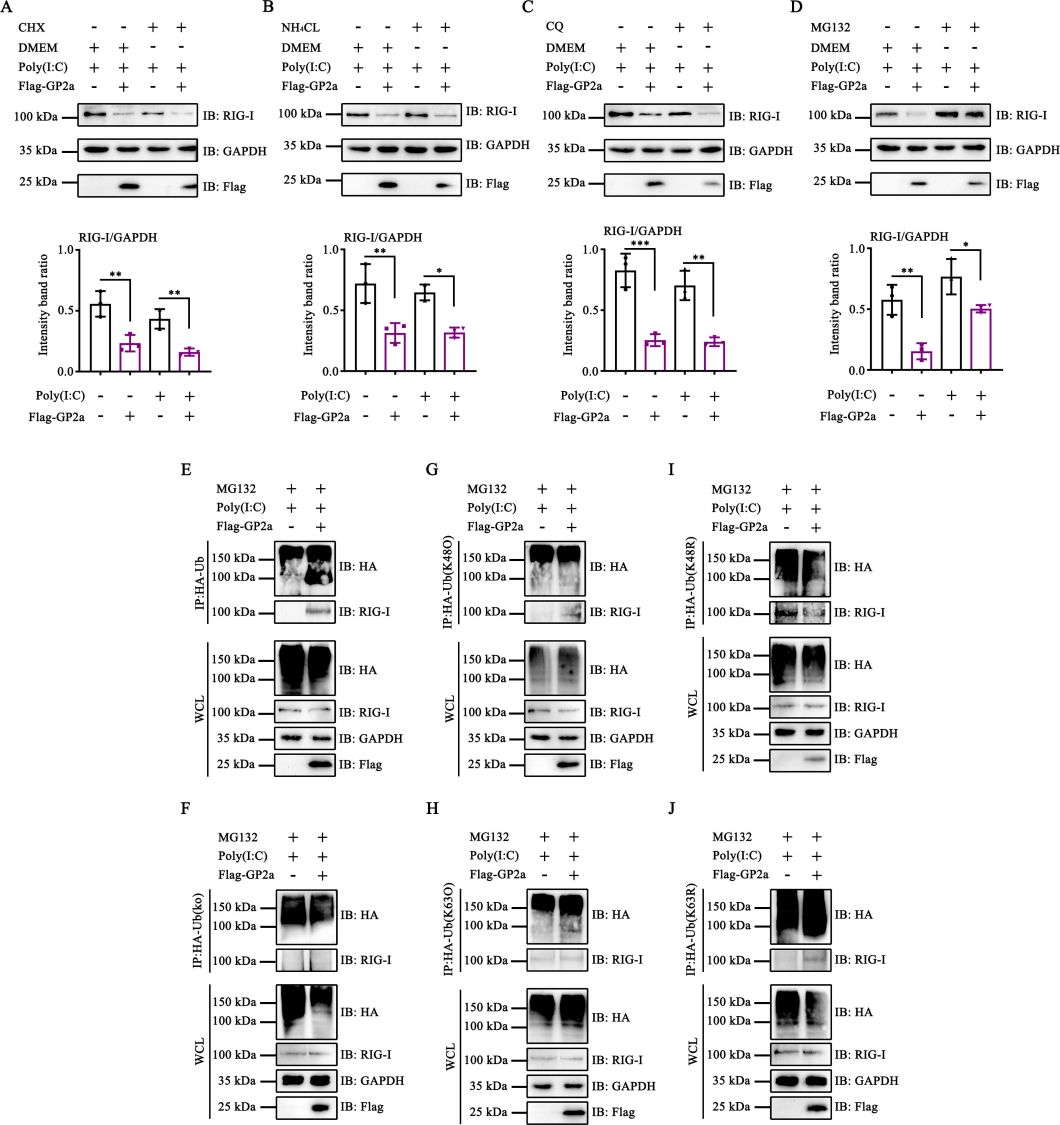
PRRSV GP2a promotes K48-linked ubiquitination of RIG-I and inhibits K63-linked ubiquitination of RIG-I. (**A–D**) HEK-293T cells were transfected with pCAGGS-3×Flag-GP2a. (**A**) DMEM and CHX (working concentration, 10 μg/mL) were added to the cells. (**B**) NH_4_Cl (working concentration, 10 µg/mL) was added to the cells. (**C**) CQ (working concentration, 5 µM) was added to the cells. (**D**) MG132 (working concentration, 5 µM) was added to the cells. (**A–D**) The cells were collected to detect RIG-I, GAPDH, and Flag using Western blot assay. (**E–J**) HEK-293T cells were co-transfected with pCAGGS-2 HA-Ub or plasmids expressing Ub mutants and pCAGGS-3×Flag-GP2a. The cells were then treated with MG132. The interaction between Ub (or Ub mutants) and RIG-I was detected using Western blot assay. (**A–D**) Relative band intensities were quantified using ImageJ software. Data are shown as mean ± SD from three independent experiments (one-way ANOVA; *, *P* < 0.05; **, *P* < 0.01; ***, *P* < 0.001). ns, not significant.

To investigate the impact of GP2a on the downregulation of RIG-I expression, HEK-293T cells were treated with cycloheximide (CHX) to inhibit RIG-I production. The RIG-I expression was decreased in GP2a-transfected cells, suggesting that GP2a plays a role in inducing the RIG-I degradation process (Fig. 3A). We then detected the GP2a-mediated RIG-I degradation pathway. Cells were treated with MG132 (a proteasome pathway inhibitor), NH_4_Cl (an autophagy pathway inhibitor), or CQ (an apoptosis pathway inhibitor). GP2a consistently induced RIG-I degradation in cells treated with NH_4_Cl and CQ (Fig. 3B and C). However, this degradation was attenuated in cells treated with MG132, implying that MG132 effectively antagonized GP2a-induced RIG-I degradation (Fig. 3D). These findings indicate that GP2a induces RIG-I degradation via the proteasomal pathway.

K63-linked ubiquitination is essential for the activation of RIG-I, whereas K48-linked ubiquitination leads to the proteasomal degradation of RIG-I (31, 32). We performed co-immunoprecipitation (Co-IP) assays to investigate whether PRRSV GP2a promoted RIG-I ubiquitination. As expected, GP2a promoted the interaction between Ub and RIG-I, suggesting that GP2a increased the ubiquitination of RIG-I (Fig. 3E and F). Subsequently, we identified the type of ubiquitination of RIG-I induced by GP2a. GP2a promoted the interaction between Ub-K48O and RIG-I, indicating that GP2a facilitates the K48-linked ubiquitination of RIG-I (Fig. 3G). Furthermore, GP2a inhibited the interaction between Ub-K63O and RIG-I, indicating that GP2a suppresses K63-linked ubiquitination of RIG-I (Fig. 3H). To validate these findings, we assessed the interaction between RIG-I and the Ub mutants (Ub-K48R and Ub-K63R). GP2a induced the interaction between Ub-K63R and RIG-I and inhibited the interaction between Ub-K48R and RIG-I (Fig. 3I and J). These results demonstrated that GP2a induces RIG-I degradation by promoting K48-linked ubiquitination and prevents RIG-I activation by inhibiting K63-linked ubiquitination.

### PRRSV GP2a affected the interaction between RIG-I and E3 Ub ligases

**Fig 4:**
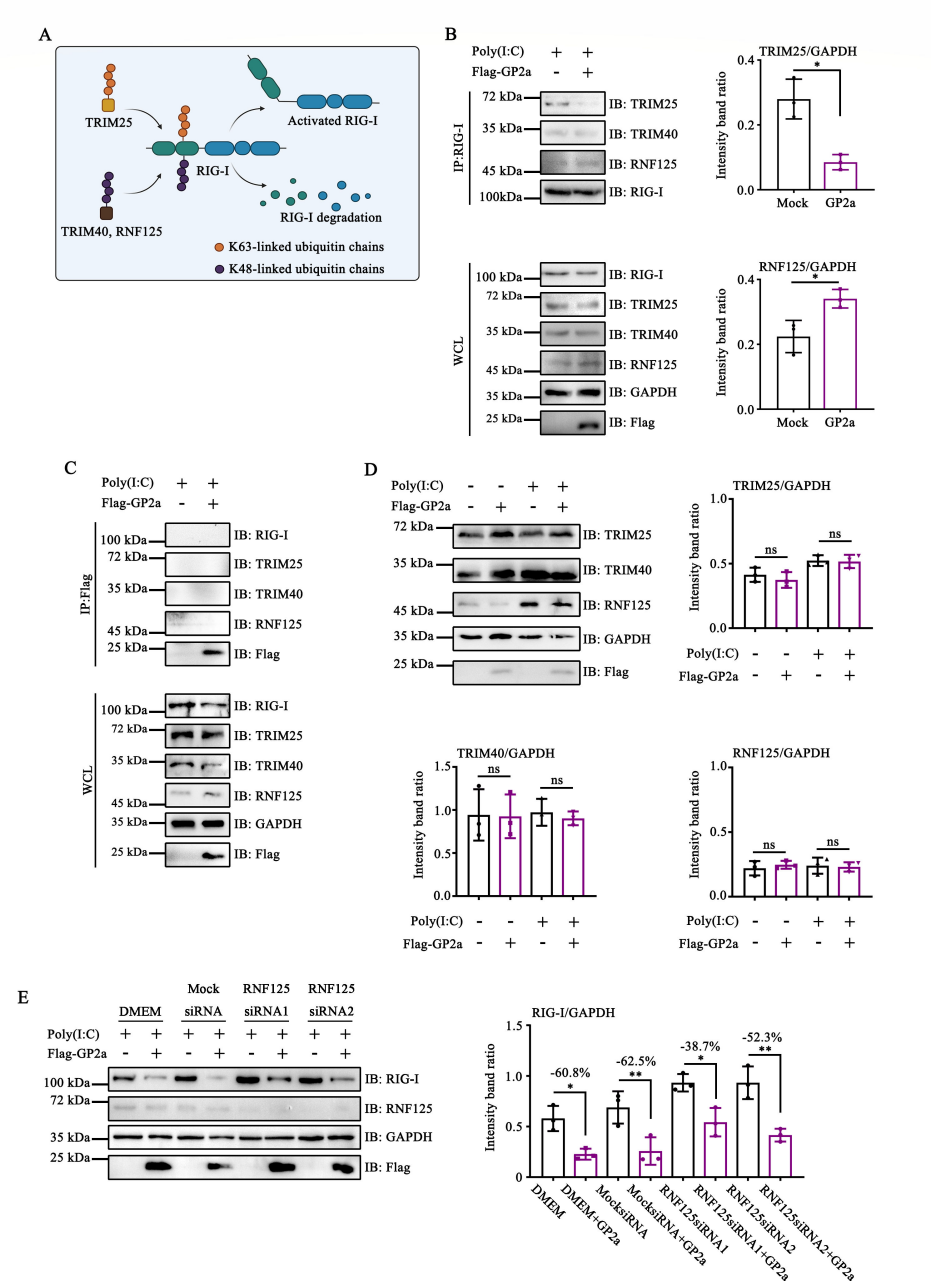
PRRSV GP2a affected the interaction between RIG-I and E3 Ub ligases. (**A**) Diagram of RIG-I ubiquitination by E3 Ub ligases. (**B and C**) HEK-293T cells were transfected with pCAGGS-3×Flag-GP2a. The interacting proteins were detected by Western blot assay. (**D**) HEK-293T cells were transfected with pCAGGS-3×Flag-GP2a. The cells were collected to detect the expressions of TRIM25, TRIM40, RNF125, GAPDH, and Flag. (**E**) HEK-293T cells were transfected with small interfering RNAs (siRNAs). After 24 h, cells were transfected with pCAGGS-3×Flag-GP2a. Cells were collected to detect the expression of RIG-I, RNF125, GAPDH, and Flag. (**B, D, and E**) Relative band intensities were quantified using ImageJ software. Data are shown as mean ± SD from three independent experiments (Student’s *t*-test for B; one-way ANOVA for D and E; *, *P* < 0.05; **, *P* < 0.01). ns, not significant.

Previous studies have demonstrated that TRIM25 plays a crucial role in the K63-linked ubiquitination of RIG-I, whereas TRIM40 and RNF125 act as negative regulatory factors that promote the K48-linked ubiquitination of RIG-I (Fig. 4A) (18–20). To further elucidate the mechanism by which GP2a interferes with RIG-I ubiquitination, we examined whether GP2a blocked the interaction between RIG-I and these E3 Ub ligases. Immunoprecipitation experiments revealed that GP2a blocked the interaction between RIG-I and TRIM25, implying that GP2a inhibited TRIM25-mediated K63-linked ubiquitination of RIG-I (Fig. 4B). Furthermore, GP2a facilitated RNF125-mediated K48-linked ubiquitination of RIG-I by promoting the interaction between RIG-I and RNF125 (Fig. 4B). Subsequent studies demonstrated that GP2a does not interact with E3 Ub ligases or RIG-I (Fig. 4C). Western blot analysis revealed that GP2a did not influence the endogenous expression of TRIM25, TRIM40, or RNF125 (Fig. 4D). In summary, these findings suggest that GP2a disrupts TRIM25-mediated ubiquitination and promotes RNF125-mediated RIG-I ubiquitination.

To further substantiate the hypothesis that GP2a hijacks RNF125 to promote RIG-I degradation, we synthesized small interfering RNAs (siRNAs) to downregulate RNF125 expression. The results showed that GP2a reduced RIG-I expression by 60.8% in the Dulbecco’s modified Eagle medium (DMEM) group and 62.5% in the mock siRNA group, whereas GP2a only decreased RIG-I expression by 38.7% and 52.3% in the RNF125-siRNA1 and RNF125-siRNA2 groups, respectively (Fig. 4E). These findings indicated that GP2a induced the degradation of RIG-I through hijacking RNF125.

### PRRSV GP2a interacted with ZCCHC3

**Fig 5:**
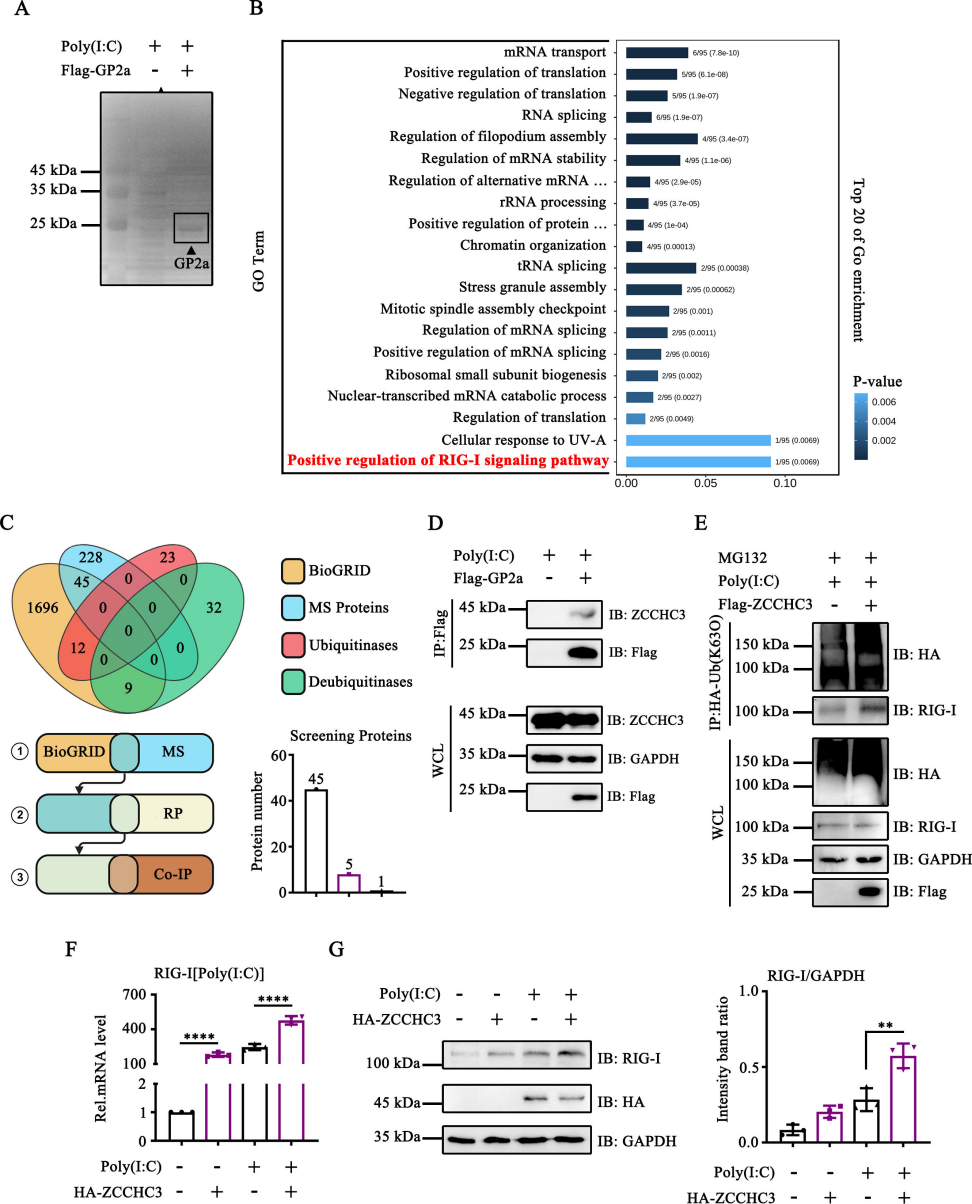
PRRSV GP2a interacted with ZCCHC3. (**A**) The GP2a enriched silver staining. (**B**) A bar plot illustrating enriched GO terms for the top 20 hits was generated using the Metascape analysis webtool. (**C**) The Venn diagram illustrates overlapping proteins in the three data sets sourced from BioGRID (https://thebiogrid.org/), Ubibrowser-E3 Ub ligase (http://ubibrowser.bio-it.cn/ubibrowser/home/index), and Ubibrowser-Deubiquitinase (http://ubibrowser.bio-it.cn/ubibrowser/home/index). (**D**) HEK-293T cells were transfected with pCAGGS-3×Flag-GP2a. The interaction between ZCCHC3 and Flag was detected using Western blot assay. (**E**) HEK-293T cells were co-transfected with pCAGGS-2×HA-Ub(K63O) and pCAGGS-3×Flag-ZCCHC3. The interaction between Ub and RIG-I was detected using Western blot assay. (**F and G**) HEK-293T cells were transfected with pCAGGS-2×HA-ZCCHC3. (**F**) The cells were collected to detect RIG-I mRNA levels. The experiment was conducted three times, and the data are shown as mean ± SD from triplicate wells in the same experiment (one-way ANOVA; ****, *P* < 0.0001). (**G**) The cells were collected to assess the expression of RIG-I, HA, and GAPDH. Relative band intensities were quantified using ImageJ software. Data are shown as mean ± SD from three independent experiments (one-way ANOVA, **, *P* < 0.01).

RIG-I ubiquitination is modulated by various host factors. We speculate that GP2a hijacks unidentified regulatory factors that influence E3 Ub ligases. To identify the potential host factors involved in this degradation process, we performed liquid chromatography-tandem mass spectrometry analysis (Fig. 5A). This analysis identified 228 potential factors that could bind to GP2a. Gene ontology (GO) enrichment analysis indicated a significant enrichment of genes within the RIG-I-like receptor (RLR) signaling pathway, suggesting that PRRSV GP2a blocks the RIG-I signaling pathway to suppress IFN production (Fig. 5B). Subsequently, we compared the mass spectrometry (MS) results with various databases to identify the potential host factors implicated in GP2a-mediated RIG-I degradation. The BioGRID database was used to screen proteins that interact with RIG-I through biological analysis. The Ubibrowser database was used to screen Ub ligases involved in RIG-I ubiquitination and deubiquitination. Additionally, the proteins reported in the literature to interact with RIG-I were systematically screened utilizing the UniProt database. A comparative analysis between the mass spectrometry results and the BioGRID database reveals an overlap of 45 proteins (Fig. 5C). These 45 proteins were further cross-referenced with the UniProt database, resulting in the identification of five proteins (Fig. 5C). Co-IP assays demonstrated that GP2a interacts with ZCCHC3 (Fig. 5D). In addition, none of the results of the mass spectrometry analysis overlapped with the Ubibrowser database, suggesting that GP2a does not directly interact with the Ub ligases (Fig. 5C).

Next, we investigated the effect of ZCCHC3 on RIG-I. ZCCHC3 promotes the interaction between RIG-I and Ub(K63O), suggesting that ZCCHC3 induces the activation of RIG-I by promoting K63-linked ubiquitination of RIG-I (Fig. 5E). Notably, ZCCHC3 upregulated RIG-I mRNA and induced RIG-I expression (Fig. 5F and G). In conclusion, these results indicate that GP2a targeted RIG-I by hijacking ZCCHC3.

### PRRSV GP2a hijacked ZCCHC3 to inhibit RIG-I activation

**Fig 6:**
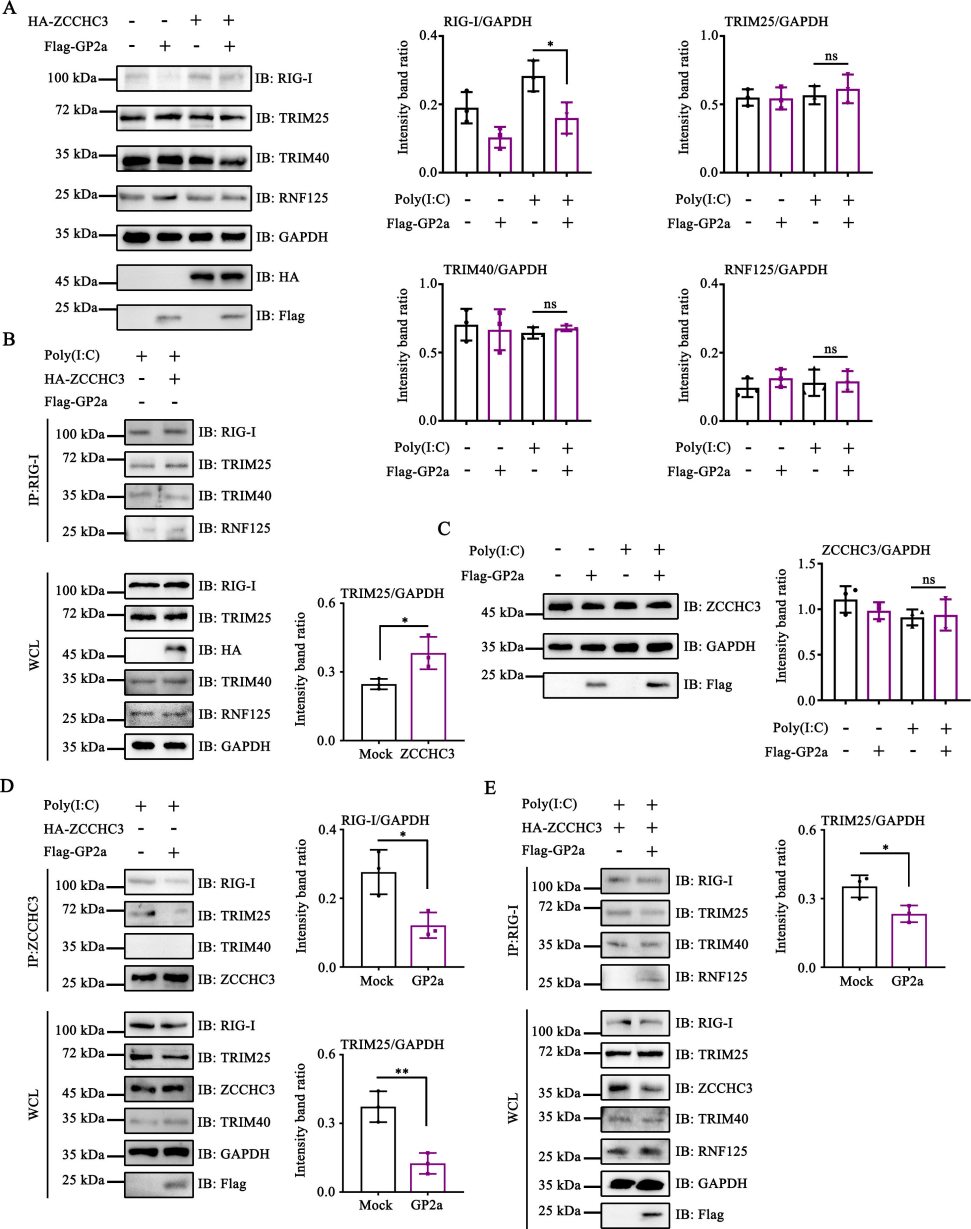
PRRSV GP2a hijacked ZCCHC3 to inhibit RIG-I activation. (**A**) HEK-293T cells were co-transfected with pCAGGS-3×Flag-GP2a and pCDNA3.1-2×HA-ZCCHC3. The cells were then collected to detect the expression of TRIM25, TRIM40, RNF125, RIG-I, GAPDH, HA, and Flag using Western blot assay. (**B**) HEK-293T cells were transfected with pCDNA3.1-2×HA-ZCCHC3. The interacting proteins were detected by Western blot assay. (**C and D**) HEK-293T cells were transfected with pCAGGS-3×Flag-GP2a. (**C**) The cells were subsequently collected to detect the expression of ZCCHC3, GAPDH, and Flag. (**D**) The interacting proteins were detected by Western blot assay. (**E**) HEK-293T cells were co-transfected with pCAGGS-3×Flag-GP2a and pCDNA3.1-2×HA-ZCCHC3. The interacting proteins were detected by Western blot assay. (**A, C, and D**) Relative band intensities were quantified using ImageJ software. Data are shown as mean ± SD from three independent experiments (one-way ANOVA for **A and C**; Student’s *t*-test for B, D, and E; *, *P* < 0.05; **, *P* < 0.01). ns, not significant.

First, we investigated the specific functions of ZCCHC3 in RIG-I activation. Our results indicated that ZCCHC3 did not affect the expression of TRIM25, TRIM40, or RNF125 (Fig. 6A). Subsequent analyses revealed that ZCCHC3 improved the interaction between TRIM25 and RIG-I, implying that ZCCHC3 facilitated TRIM25-mediated ubiquitination of RIG-I (Fig. 6B). Furthermore, ZCCHC3 did not alter the interactions between TRIM40, RNF125, and RIG-I, suggesting that it does not affect TRIM40- and RNF125-mediated RIG-I degradation (Fig. 6B). Notably, GP2a-mediated RIG-I degradation was reduced in the ZCCHC3 overexpressing cells, underscoring the critical role of ZCCHC3 in this degradation pathway (Fig. 6A).

Subsequently, we examined the inhibitory effect of GP2a on ZCCHC3-mediated RIG-I activation. Our results demonstrate that GP2a did not suppress ZCCHC3 expression (Fig. 6C). Immunoprecipitation assays revealed that ZCCHC3 interacted with TRIM25 and RIG-I, whereas GP2a disrupted this interaction (Fig. 6D). These findings indicated that GP2a is associated with ZCCHC3, which impedes the formation of the ZCCHC3-TRIM25-RIG-I complex. Further analysis revealed that GP2a blocks the ZCCHC3-promoted interaction between TRIM25 and RIG-I. (Fig. 6E). In summary, these results indicate that GP2a inhibits TRIM25-mediated RIG-I activation by targeting ZCCHC3.

### PRRSV GP2a hijacked ZCCHC3 to inhibit RIG-I production

**Fig 7:**
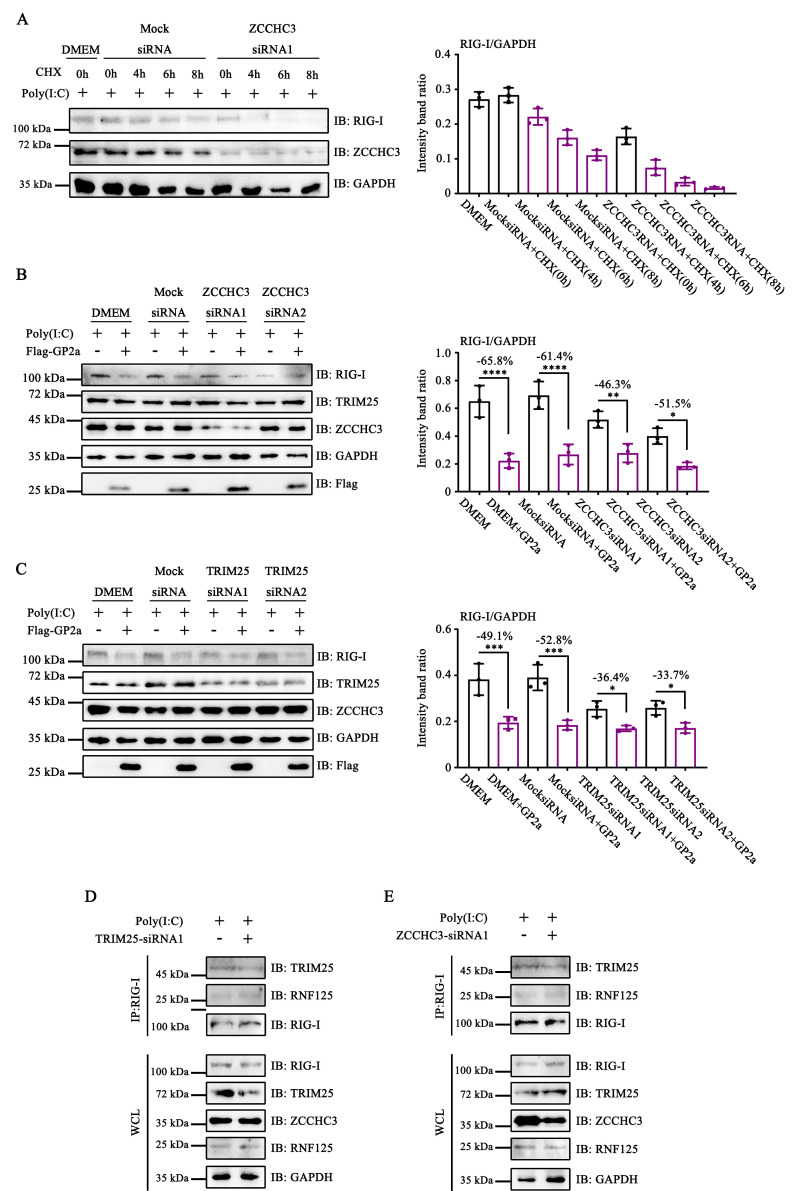
PRRSV GP2a hijacked ZCCHC3 to inhibit RIG-I production. (**A**) HEK-293T cells were transfected with siRNAs. Cells were collected to detect the expression of RIG-I, ZCCHC3, and GAPDH. (**B–E**) HEK-293T cells were transfected with siRNAs. After 24 h, cells were transfected with pCAGGS-3×Flag-GP2a. (**B and C**) Cells were collected to detect the expression of TRIM25, RIG-I, ZCCHC3, GAPDH, and Flag. (**A–C**) Relative band intensities were quantified using ImageJ software. Data are shown as mean ± SD from three independent experiments (one-way ANOVA; *, *P* < 0.05; **, *P* < 0.01; ***, *P* < 0.001; ****, *P* < 0.0001). (**D and E**) The interacting proteins were detected by Western blot assay.

Subsequently, we investigated the potential inhibitory effects of GP2a on ZCCHC3-mediated RIG-I production. To exclude the possibility of RIG-I degradation due to ZCCHC3 knockdown, CHX was administered to the cells, and samples were collected at various time points. The rate of RIG-I degradation in ZCCHC3 knockdown cells was comparable to that of the negative control, indicating that ZCCHC3 knockdown does not contribute to RIG-I degradation (Fig. 7A). Notably, ZCCHC3 knockdown leads to a decrease in RIG-I expression, implying that ZCCHC3 is crucial for RIG-I production (Fig. 7A).

The inhibition of K63-linked ubiquitination of RIG-I effectively impedes the RLR signaling pathway, thereby suppressing the production of ISGs and ultimately diminishing RIG-I production. We subsequently explored whether GP2a suppressed RIG-I production by interacting with ZCCHC3. Notably, in the DMEM and mock siRNA groups, GP2a reduced RIG-I expression by 64.1% and 62.7%, respectively. Conversely, in ZCCHC3-siRNA1 and ZCCHC3-siRNA2 groups, GP2a decreased RIG-I expression by only 45.8% and 44.7%, respectively (Fig. 7B). These findings suggest that GP2a inhibits RIG-I production, whereas ZCCHC3 knockdown impairs the ability of GP2a to suppress RIG-I production.

We hypothesized that ZCCHC3 induces RIG-I production by facilitating TRIM25-mediated RIG-I activation, whereas GP2a hijacks ZCCHC3 to inhibit RIG-I production. To verify this hypothesis, we synthesized siRNAs to downregulate TRIM25 expression. GP2a reduced RIG-I expression by 45.5% and 49.2% in the DMEM and mock siRNA groups, respectively, whereas GP2a only decreased RIG-I expression by 38.1% and 38.5% in the TRIM25-siRNA1 and TRIM25-siRNA2 groups, respectively (Fig. 7C). These findings indicated that GP2a hijacked ZCCHC3 to inhibit TRIM25-mediated RIG-I activation, thereby preventing RIG-I production.

Furthermore, immunoprecipitation experiments were performed to exclude the possibility that the knockdown of TRIM25 and ZCCHC3 led to RNF125-mediated RIG-I degradation. Knockdown of TRIM25 and ZCCHC3 did not enhance the interaction between RNF125 and RIG-I. This result suggests that the function of GP2a hijacking of ZCCHC3 to block RIG-I activation and production is not associated with its promotion of RNF125-mediated RIG-I degradation (Fig. 7D and E).

### Identifying the key amino acid sites of GP2a that induce RIG-I degradation

**Fig 8:**
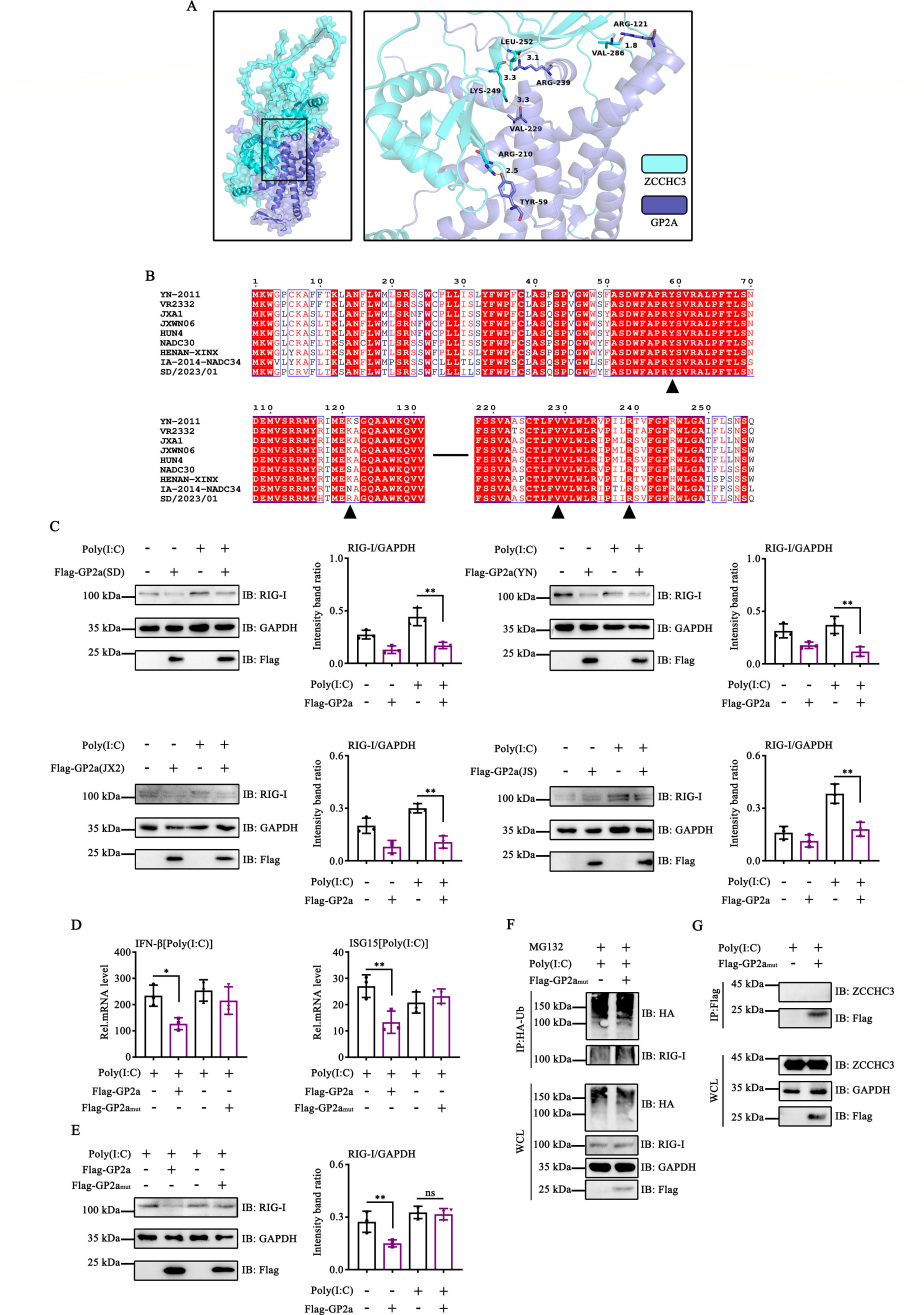
Identification of key amino acid sites of GP2a that induce RIG-I degradation. (**A**) Schematic illustration of molecular docking. (**B**) Schematic illustration of multiple GP2a sequence alignment. (**C**) HEK-293T cells were transfected with plasmids expressing the different PRRSV GP2a strains. The cells were collected to detect the expression of RIG-I, GAPDH, and Flag. (**D and E**) HEK-293T cells were transfected with either pCAGGS-3×Flag-GP2a or pCAGGS-3×Flag-GP2a_mut_. (**D**) After 24 h, cells were stimulated with Poly(I:C) for 12 h. The cells were collected to detect IFN-β and ISG15 mRNA levels using RT-qPCR. The experiment was conducted three times, and the data are shown as mean ± SD from triplicate wells in the same experiment (one-way ANOVA; *, *P* < 0.05; **, *P* < 0.01). (**E**) The cells were collected to detect the expression of RIG-I, GAPDH, and Flag. (**F and G**) The HEK-293T cells were transfected with pCAGGS-3×Flag-GP2a_mut_. The interacting proteins were detected by Western blot assay. (**C and E**) Relative band intensities were quantified using ImageJ software. Data are shown as mean ± SD from three independent experiments (one-way ANOVA; **, *P* < 0.01). ns, not significant.

The predicted structures of GP2a and ZCCHC3 were generated using AlphaFold to identify the critical amino acids in GP2a that facilitate its interaction with ZCCHC3. Protein-protein docking was performed using the Docking Web Server (GRAMM), and the interaction between GP2a and ZCCHC3 was predicted and visualized using PyMOL. The results indicated that Tyr59, Arg121, Val229, and Arg239 were essential residues for the binding of GP2a to ZCCHC3 (Fig. 8A). Sequence alignment analysis revealed that these amino acids were conserved across different strains, indicating that the function of GP2a in interacting with ZCCHC3 is conserved (Fig. 8B). To confirm this result, we analyzed whether the GP2a proteins of different PRRSV strains had the same effect and found that they all inhibit RIG-I expression (Fig. 8C). To evaluate the functional significance of these conserved amino acid sites, they were mutated to alanine to generate a plasmid encoding GP2a_mut_. As expected, GP2a_mut_ did not inhibit IFN-β and ISG15 mRNA expression stimulated by Poly(I:C) (Fig. 8D). The ability of GP2a_mut_ to inhibit RIG-I production was weakened (Fig. 8E). GP2a_mut_ did not promote RIG-I ubiquitination (Fig. 8F). Immunoprecipitation experiments revealed that GP2a_mut_ did not interact with ZCCHC3 (Fig. 8G). Collectively, these findings underscore the significance of Tyr59, Arg121, Val229, and Arg239 as crucial residues for the hijacking of ZCCHC3 by GP2a.

### GP2a inhibits the function of ZCCHC3 in suppressing PRRSV replication

**Fig 9:**
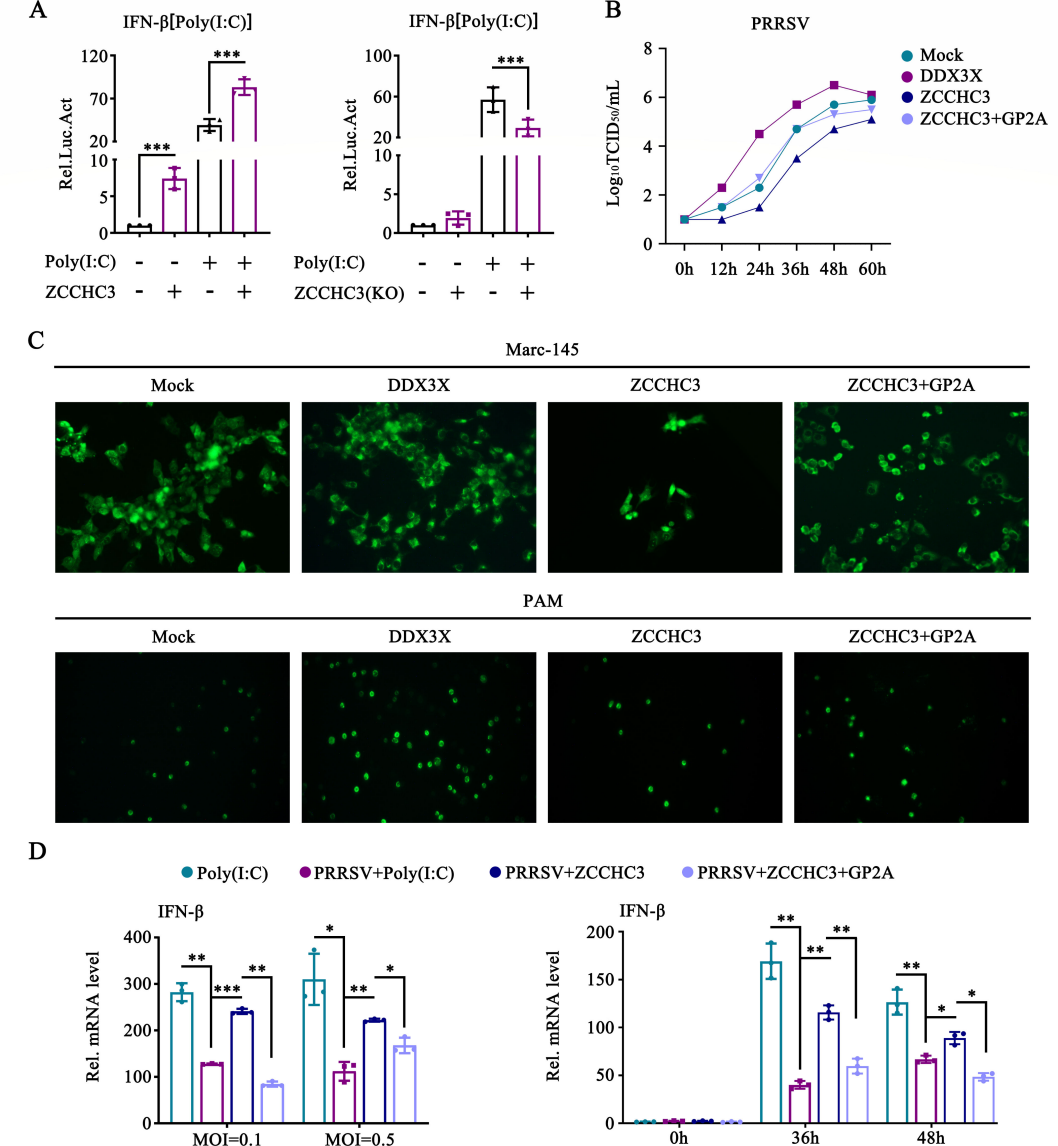
GP2a inhibits the function of ZCCHC3 in suppressing PRRSV replication. (**A**) HEK-293T cells were co-transfected with siRNA or pCAGGS-2×HA-ZCCHC3, pIFN-β-luc, and pRL-TK. The cells were collected to detect IFN-β promoter activity using a dual-luciferase reporter assay. (**B**) Marc-145 cells were co-transfected with pCAGGS-3×Flag-GP2a, pCAGGS-2×HA-ZCCHC3, and pCAGGS-2×HA-DDX3X. The cell supernatants were collected to detect the TCID_50_. The experiment was conducted three times. (**C**) Marc-145 and PAM cells were infected with PRRSV at an MOI of 0.1 for 36 h. The cells were stained with PRRSV N mouse-pAb, followed by Alexa Fluor 488-conjugated goat antimouse staining (green). The nuclei were stained with 4′,6-diamidino-2-phenylindole (blue). (**D**) Marc-145 cells were co-transfected with pCAGGS-3×Flag-GP2a and pCAGGS-2×HA-ZCCHC3. The cells were infected with PRRSV and collected to detect the IFN-β mRNA level using RT-qPCR. (**A and D**) The experiment was conducted three times, and the data are shown as mean ± SD from triplicate wells in the same experiment (one-way ANOVA; *, *P* < 0.05; **, *P* < 0.01; ***, *P* < 0.001).

We investigated the effect of ZCCHC3 on IFN activity. The results demonstrate that ZCCHC3 overexpression significantly promoted the production of IFN-β, whereas the levels of IFN-β were reduced in ZCCHC3 knockdown cells (Fig. 9A). We further investigated the role of ZCCHC3 in PRRSV replication. DDX3X, a known facilitator of PRRSV replication, was used as the positive control (33). DDX3X increased the PRRSV titer during the early stages of infection, whereas ZCCHC3 significantly decreased the PRRSV titer (Fig. 9B). Notably, GP2a inhibited the ability of ZCCHC3 to suppress PRRSV replication (Fig. 9B). Our findings were further validated using the immunofluorescence assay (IFA) results (Fig. 9C). Moreover, ZCCHC3 inhibited PRRSV-induced suppression of the IFN response. This inhibition was diminished in the cells overexpressing GP2a (Fig. 9D). In summary, our findings suggest that ZCCHC3 acts as an antiviral factor against PRRSV, whereas GP2a counteracts this antiviral function.

## DISCUSSION

Viruses have developed sophisticated strategies to evade innate immune responses in the host. One of the most efficient strategies is the inhibition of IFN production by targeting RIG-I. Human papillomavirus E6 protein promotes K48-linked ubiquitination of TRIM25, leading to its degradation and inhibition of TRIM25-mediated activation of RIG-I (34). Nonstructural protein 5 of the severe acute respiratory syndrome coronavirus 2 (SARS-CoV-2) cleaves RIG-I to disrupt the formation of the RIG-I-MAVS complex (35). Besides, the SARS-CoV-2 N protein interacts with G3BP1 to block the G3BP1-mediated degradation of RNF125, thereby inducing the degradation of RIG-I (35). The foot-and-mouth disease virus 2B protein recruits RNF125 to promote the degradation of RIG-I (36). Similarly, PRRSV has evolved strategies to disrupt the IFN system by targeting RIG-I. Nsp1α and N protein hijack TRIM25 to inhibit TRIM25-mediated activation of RIG-I (25, 28). Nsp2 interacts with SH3KBP1 to induce autophagic degradation, ultimately inhibiting the K63-linked ubiquitination of RIG-I (21). Nsp11 inhibits the expression of RIG-I (37). In this study, we observed that PRRSV significantly induced RIG-I degradation, with GP2a playing a critical role in this process. Further studies have revealed that GP2a mediates RIG-I degradation via the Ub-proteasome pathway. Specifically, GP2a enhances the K48-linked ubiquitination of RIG-I by promoting the interaction between RIG-I and RNF125. Notably, GP2a did not directly bind to RNF125, implying that GP2a modulates the interaction between RNF125 and RIG-I through indirect mechanisms. It is imperative to elucidate the specific mechanisms involved in the GP2a-induced RIG-I degradation.

RIG-I activation is dependent on TRIM25-mediated K63-linked ubiquitination, which is crucial for initiating the innate immune response (20). In this study, we observed that ZCCHC3 recruits TRIM25 to facilitate the formation of the TRIM25-RIG-I complex, thereby increasing the K63-linked ubiquitination of RIG-I. Furthermore, ZCCHC3 did not affect the interaction between RIG-I and either RNF125 or TRIM40, indicating that ZCCHC3 did not interfere with RIG-I degradation. PRRSV GP2a did not affect ZCCHC3 expression. Subsequent studies have revealed that GP2a hijacked ZCCHC3 to inhibit RIG-I activation. Specifically, GP2a interacted with ZCCHC3 to disrupt the interaction between ZCCHC3 and TRIM25. This disruption inhibits TRIM25-mediated K63-linked ubiquitination of RIG-I, thereby preventing RIG-I activation.

IFN-β is believed to facilitate RIG-I production through a positive feedback mechanism (18, 38). Numerous studies have highlighted the importance of this feedback loop. The upregulation of IFN-induced RIG-I expression subsequently induces the activation of antiviral proteins and the production of IFN (38, 39). This regulatory mechanism ensures that upon detection of viral infection using RIG-I, the IFN system is rapidly activated to inhibit viral propagation (38, 39). In this study, we observed that the knockdown of TRIM25 and ZCCHC3 resulted in a reduction in RIG-I production, indicating that K63-linked ubiquitination is crucial for RIG-I production. ZCCHC3 induces IFN production by enhancing TRIM25-mediated K63-linked ubiquitination of RIG-I. Subsequently, IFN activates the JAK-STAT signaling pathway to induce the expression of ISGs, ultimately promoting RIG-I production. PRRSV GP2a hijacked ZCCHC3 to inhibit K63-linked ubiquitination of RIG-I, disrupting the RIG-I positive feedback loop and consequently inhibiting RIG-I production. Collectively, these findings suggested that GP2a hijacked ZCCHC3 to reduce RIG-I production.

PRRSV is characterized by its high genetic variability, and the GP2a sequence exhibits divergence among various PRRSV strains (40). In this study, we used GRAMM software to conduct docking analyses between GP2a and ZCCHC3, identifying Tyr59, Arg121, Val229, and Arg239 as critical residues for the binding interaction. Notably, these key amino acid residues were conserved across different PRRSV strains, indicating that NADC30-like, classical, and highly pathogenic strains of PRRSV GP2a play similar roles in RIG-I degradation. Additionally, ZCCHC3 significantly enhanced IFN production, thereby inhibiting PRRSV replication. ZCCHC3 overexpression counteracted PRRSV-induced reduction in IFN-β and ISG15 expression, whereas GP2a diminished this function. These results indicate that ZCCHC3 functions as an antiviral factor against PRRSV, while GP2a counteracts this antiviral activity.

**Fig 10:**
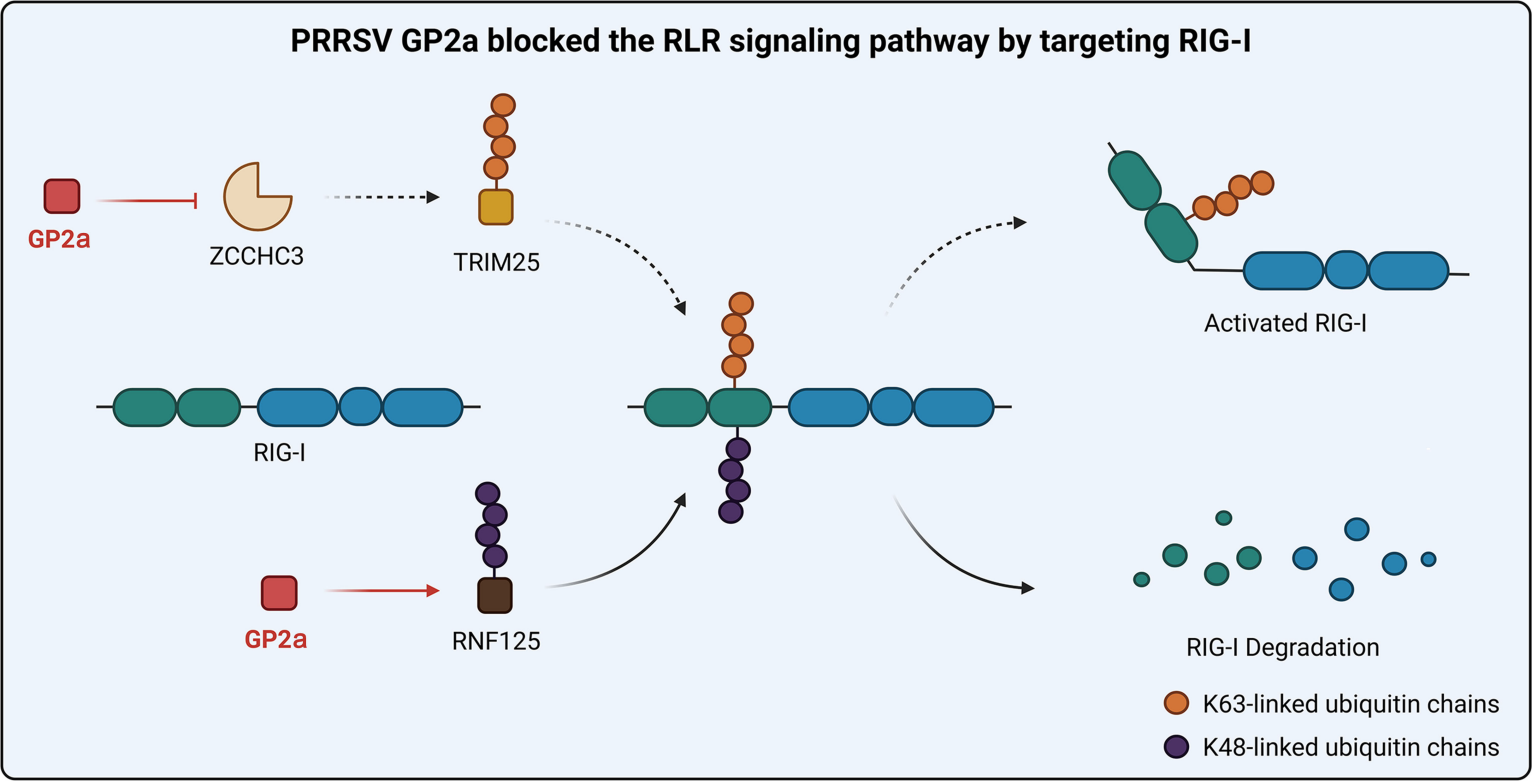
PRRSV GP2a inhibits the RLR signaling pathway by targeting RIG-I. PRRSV GP2a promotes the interaction between RNF125 and RIG-I, leading to RIG-I degradation. GP2a hijacked ZCCHC3 to inhibit K63-linked ubiquitination and RIG-I expression.

In conclusion, this study demonstrates that PRRSV GP2a inhibits RIG-I expression through two mechanisms: (i) PRRSV GP2a facilitates the K48-linked ubiquitination of RIG-I by improving the interaction between RIG-I and RNF125, thereby promoting the degradation of RIG-I, and (ii) GP2a hijacks ZCCHC3 to inhibit TRIM25-mediated activation of RIG-I, thereby suppressing IFN production and ultimately inhibiting RIG-I production (Fig. 10). This study fills a gap in our understanding of the mechanisms by which PRRSV GPs block the IFN system. It is imperative to understand these mechanisms at the molecular level to develop effective strategies to combat PRRSV infections.

## MATERIALS AND METHODS

### Virus, cells, and plasmids

The PRRSV-JX02 strain, PRRSV-JS strain, PRRSV-SD strain, PRRSV-YN strain, VSV-GFP, and SeV were stored in our laboratory. HEK-293T, pulmonary alveolar macrophage (PAM), and African green monkey kidney epithelial cell line (Marc-145) cells were cultured in DMEM supplemented with 10% fetal bovine serum and 1% penicillin-streptomycin and incubated at 37°C with 5% CO_2_. Full-length cDNAs encoding PRRSV structural proteins were amplified from the PRRSV cDNA and cloned into the pCAGGS-3×Flag vector. Full-length cDNAs encoding ZCCHC3 and DDX3X were amplified from HEK-293T cDNA and cloned into the pCAGGS-2×HA vector. The pIFN-β-luc, pRL-TK, and plasmids expressing Ub were stored in our laboratory.

### Antibodies and reagents

The following antibodies were used in this study: RIG-I/DDX58 rabbit mAb (A22478), MDA5 rabbit mAb (A2419), NF-κB p65/RelA rabbit mAb (A19653), phospho-NF-κB p65/RelA-S536 rabbit mAb (AP1294), IRF7 rabbit pAb (A0159), IRF3 rabbit mAb (A19717), phospho-IRF3-S396 rabbit mAb (AP1412), DDDDK-tag rabbit mAb (AE092), GAPDH mouse mAb (AC002), ZCCHC3 rabbit pAb (A17235), TRIM25 rabbit mAb (A25846), HRP-conjugated rabbit antigoat IgG (H + L) (AS029), and HRP-conjugated goat anti-mouse IgG (H + L) (AS003) from ABclonal Biotechnology; phospho-IRF-7 (Ser471/472) antibody (5184) from CST; and TRIM40 monoclonal antibody (67073) and RNF125 polyclonal antibody (13290) from Proteintech. Dylight-conjugated 488 goat anti-mouse IgG (A23210) was obtained from Abbkine. The PRRSV-N mouse polyclonal antibodies were kept in our laboratory. RIPA lysis buffer (P0013D), MG132 (Y210207), NH4Cl (ST2030), and Z-VAD-FMK (Y062551) were obtained from Beyotime. CHX710 (HY-112951) was procured from MedChemExpress. Poly(I:C) LMW (31852-29-6) was obtained from InvivoGen. Lipofectamine 3000 (L3000015) was obtained from Thermo Fisher Scientific. Protein A/G magnetic beads (B23202) were obtained from SelleckChem. Anti-Flag M2 magnetic beads (M8823) and anti-HA magnetic beads (SAE0197) were obtained from Sigma.

### ELISA assay

For the viral infection experiment, Marc-145 cells were seeded in 24-well plates overnight. The cells were infected with PRRSV at an MOI of 0.02. The medium was replaced at 2 hpi, and the supernatants were collected at 0, 12, 24, 36, 48, 60, and 72 hpi. For the plasmid transfection experiment, HEK-293T cells were cultured overnight in 12-well plates. The cells were transfected with plasmids expressing PRRSV structural proteins at a concentration of 1,000 ng/well. After 24 h, the cells were incubated with Poly(I:C) (1,000 ng/mL) for 12 h. Supernatants were then collected, and ELISA assays were performed using an ELISA kit (BOSTER, EK2286) according to the manufacturer’s instructions.

### Virus growth kinetics

The Marc-145 cells were cultured overnight in 12-well plates. The cells were then transfected with pCAGGS-3×Flag (Mock group), pCAGGS-2×HA-DDX3X + pCAGGS-3×Flag (DDX3X group), pCAGGS-2×HA-ZCCHC3 + pCAGGS-3×Flag (ZCCHC3 group), and pCAGGS-2×HA-ZCCHC3 + pCAGGS-3×Flag-GP2a (ZCCHC3 + GP2 a group), respectively. At 24 h post-transfection, the cells were infected with PRRSV at an MOI of 0.02. The medium was replaced at 2 hpi, and the supernatants were collected at 0, 12, 24, 32, 48, and 60 hpi. Virus titers were calculated as log10 50% tissue culture infectious dose using the Reed-Muench method.

### Western blot assay

Marc-145 cells were seeded in 12-well plates and incubated overnight for viral infection experiments. The cells were infected with PRRSV at an MOI of 0.1. After 48 h, cell samples were lysed, sonicated, and centrifuged. Subsequently, the samples were combined with the loading buffer and heated for 10 min. The cell samples were then subjected to sodium dodecyl sulfate-polyacrylamide gel electrophoresis and transferred onto polyvinylidene difluoride membranes (Millipore). Membranes were blocked with 7.5% skim milk in Tris-buffered saline with 0.1% Tween 20 detergent and incubated with primary antibodies overnight at 4°C. Following this, the membranes were incubated with secondary antibodies for 2 h at room temperature and treated with enhanced chemiluminescence (Thermo Fisher Scientific). The protein bands were visualized using the Tanon imaging system (Biotanon) and analyzed using the ImageJ software. For the plasmid transfection experiment, HEK-293T cells were seeded in six-well plates overnight. The cells were then transfected with plasmids. After 24 h, the cells were stimulated with SeV or Poly(I:C) for 12 h. Cell samples were then collected, and subsequent procedures were conducted as described previously.

### VSV-GFP interferon bioassay

HEK-293T cells were cultured overnight in 12-well plates. The cells were then transfected with pCAGGS-3×Flag-GP2a. At 24 h, the cells were stimulated with Poly(I:C) for 12 h. Supernatants from the transfected cells were diluted twofold and added to fresh HEK-293T cells. The cells were then infected with VSV-GFP (MOI = 0.1), and expression was examined using inverted fluorescence microscopy.

### Co-IP assay

HEK-293T cells were seeded in six-well plates overnight and transfected with plasmids. After 24 h, the cells were stimulated with Poly(I:C) for 12 h. Cell lysates were collected using RIPA lysis buffer and centrifuged. Subsequently, the samples were incubated with anti-Flag M2 or anti-HA magnetic beads. The beads were washed thrice with RIPA lysis buffer and then treated with SDS loading buffer. The samples were then analyzed using Western blot assay. For the endogenous Co-IP experiments, HEK-293T cells were transfected with pCAGGS-3×Flag-GP2a. After 24 h, the cells were stimulated with Poly(I:C) for 12 h. Cell samples were collected using RIPA lysis buffer, centrifuged, and incubated with anti-RIG-I mAb or anti-ZCCHC3 mAb. The samples were then incubated with Protein A/G magnetic beads for 1 h at room temperature. The subsequent procedures were performed as previously described.

### Luciferase reporter gene assay

HEK-293T cells were seeded into 24-well plates and incubated overnight. The cells were then co-transfected with plasmids expressing RIG-I/MAVS (500 ng), pIFN-β-luc (100 ng), pRL-TK (25 ng), or pCAGGS-3×Flag-GP2a (200 ng). At 24 h post-transfection, the cells were collected to detect promoter activity using a dual-luciferase reporter assay. Data are expressed as the mean ± standard deviation from three independent experiments.

### Relative quantitative real-time PCR

For the viral infection experiment, Marc-145 cells were cultured overnight in 12-well plates. The cells were infected with PRRSV at an MOI of 0.1 or 0.5 for 36 h and at an MOI of 0.1 for 0, 36, or 48 h. The cells were then collected. RNA was extracted from the samples using an RNA Easy Fast Tissue/Cell Kit, treated with DNase I to remove genomic DNA, and converted to cDNA via reverse transcription PCR. IFN-β and ISG15 mRNA levels were analyzed using relative quantitative real-time PCR (RT-qPCR) with the LineGene9600 RT-PCR system. To determine whether ZCCHC3 inhibits PRRSV-mediated suppression of the IFN response, Marc-145 cells were cultured in 12-well plates overnight. The cells were then transfected with pCAGGS-3×Flag (Mock group), pCAGGS-2×HA-DDX3X + pCAGGS-3×Flag (DDX3X group), pCAGGS-2×HA-ZCCHC3 + pCAGGS-3×Flag (ZCCHC3 group), and pCAGGS-2×HA-ZCCHC3 + pCAGGS-3×Flag-GP2a (ZCCHC3 + GP2 a group), respectively. At 24 h post-transfection, the cells were infected with PRRSV at an MOI of 0.1. The cell samples were then collected, and subsequent procedures were conducted as described previously. In the plasmid transfection experiment, HEK-293T cells were cultured in 12-well plates overnight. The cells were transfected with plasmids expressing PRRSV structural proteins at a concentration of 1,000 ng/well. After 24 h, the cells were incubated with Poly(I:C) (1,000 ng/mL) for 12 h. Cell samples were then collected, and subsequent procedures were performed as described previously.

### RNA interference experiments

To exclude the possibility of RIG-I degradation due to ZCCHC3 knockdown, HEK-293T cells were cultured overnight in 12-well plates. Cells were transfected with siRNA at a concentration of 30 pm/well. After 48 h, the cells were treated with CHX. Cells were collected for Western blot analysis. For ZCCHC3 and TRIM25 knockdown experiments, HEK-293T cells were cultured in 12-well plates overnight. Cells were transfected with siRNA at a concentration of 30 pm/well. After 24 h, cells were transfected with pCAGGS-3×Flag-GP2a at 2,500 ng/well. After 24 h, the cells were stimulated with Poly(I:C) for 12 h and collected for Western blot analysis.

### Molecular docking experiment

GP2a and ZCCHC3 structures were predicted using Alphafold (version 3.0, https://alphafold.com/) and prepared using AutoDockTools (version 1.5.7, https://autodocksuite.scripps.edu/adt/) by removing water and adding polar hydrogen. GRAMM (https://gramm.compbio.ku.edu/) was used for protein-protein docking, and the resulting complex was optimized similarly. PyMOL (https://pymol.org/) was used to predict interactions and create a visualization diagram: GP2a is presented as a slate cartoon, whereas ZCCHC3 is presented as a cyan cartoon, and the binding sites are depicted in matching stick structures.

### MS analysis

Protein digestion was performed according to the filter-aided sample preparation procedure. Experiments were performed using a Q Exactive HF-X mass spectrometer coupled to an Easy nLC (Thermo Fisher Scientific). MS data were analyzed using MaxQuant software (version 1.3.0.5). The data were searched against the UniProtKB *Homo sapiens* database. The initial search was performed using a precursor mass window of 6 ppm. This search followed the enzymatic cleavage rule for trypsin. Carbamidomethyl (C) was defined as a fixed modification, whereas oxidation (M) and phosphorylation (S/T/Y) were defined as variable modifications for database searching. The cutoff global false discovery rate for peptide and protein identification was 0.01.

### IFA

Marc-145 and PAM cells were infected with PRRSV. The cells were washed thrice using phosphate-buffered saline, fixed in 4% paraformaldehyde, permeabilized with 0.1% Triton X-100, and blocked with 2% bovine serum albumin. The cells were incubated overnight with the primary antibody at 4°C and then incubated for 2 h with the secondary antibody at 37°C. All statistical experiments were performed in triplicate.

### Statistical analysis

Statistical analyses were conducted using Student’s *t*-test to analyze the two groups of data. Furthermore, statistical analyses were conducted using one-way analysis of variance (ANOVA) to analyze the multiple groups of data. Differences were considered statistically significant at *P < 0.05, **P < 0.01, and ***P < 0.001.

## Data Availability

All data are available from the corresponding author upon reasonable request.
